# Supplementary material for: Gut Microbiota Characteristics of People with Obesity by Meta-Analysis of Existing Datasets
Source: Nutrients. 2022 Jul 21;14(14):2993. doi: 10.3390/nu14142993 (PMC9325184; doi:10.3390/nu14142993)
Supplement: Supplementary file 1 [file nutrients-14-02993-s001.zip › Figure S1-11.pdf]

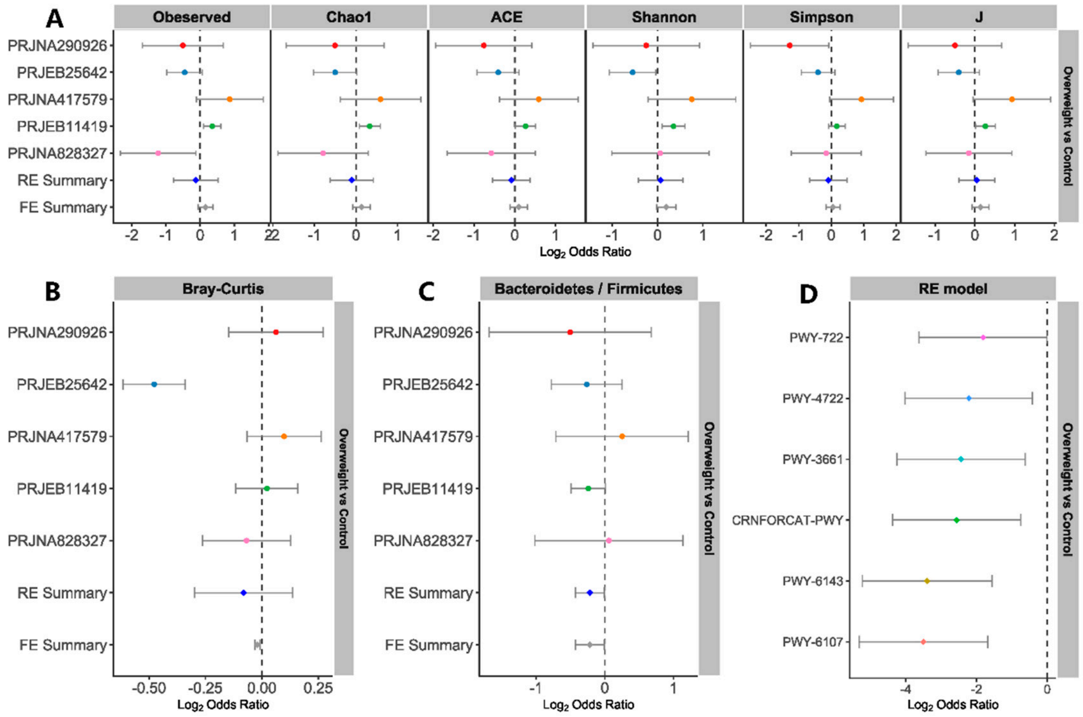

Figure S1. Comparison of bacterial alpha-diversity, beta-diversity, the Bacteroidetes / Firmicutes ratio, the pathway metrics between the overweight and control groups. Forest plots of (A) the alpha-diversity metrics, (B) Bray–Curtis distances, (C) the Bacteroidetes / Firmicutes ratio, and (D) the pathway metrics between the overweight and control groups. The error bars describe the 95% confidence intervals. A value less than 1.0 (the left of the dotted lines) indicates that the metric is higher in the overweight group than in the control group. The values larger than 1.0 (the right of the dotted lines) indicate that the metric is lower in the overweight group than in the control group. If the dotted line and the error bars did not cross, there is a significant difference between the overweight and control groups.

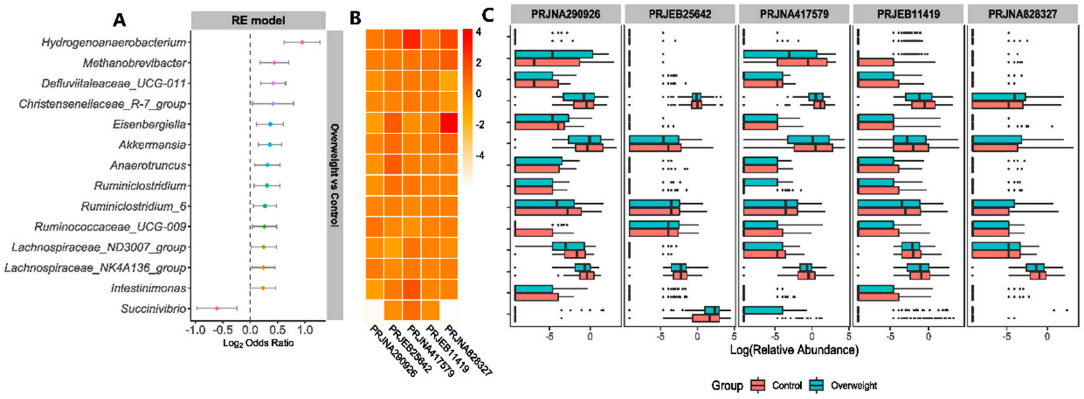

Figure S2. Discriminative taxa between overweight and control group. Forest plot (A), fold changes of control to overweight (B), and relative abundances (C) of genera with significant ORs obtained by the random effects model analysis between the overweight and control groups. The color block values greater than 0 indicates that the genera were enriched in the control groups. Log transformation is applied to fold changes and relative abundance.

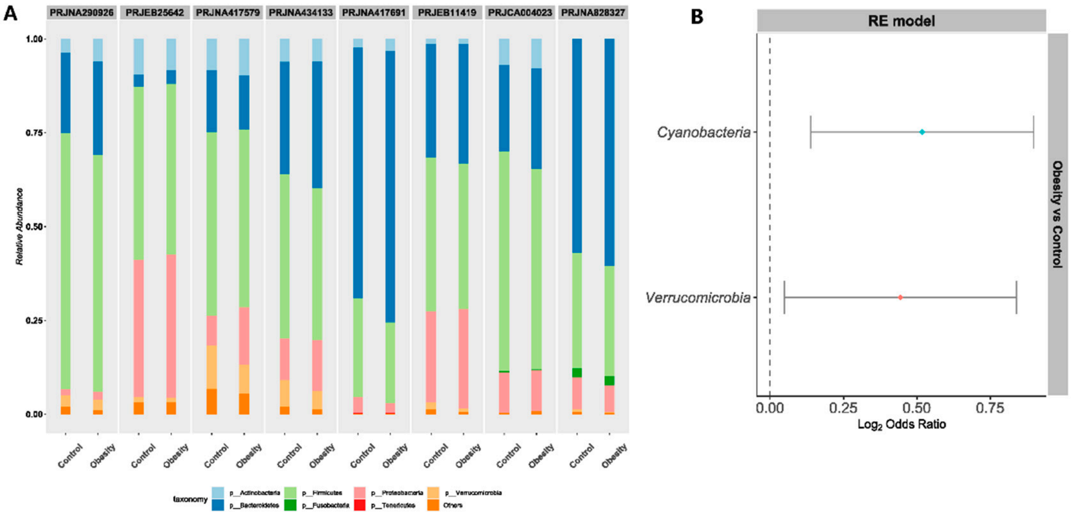

Figure S3. The top five taxa and forest plot at the phylum level between the obesity and control groups. (A) The top five taxa in terms of relative abundance at the phylum levels in each study. (B) Forest plot of phyla with significant ORs obtained by the random effects model analysis between the obesity and control groups.

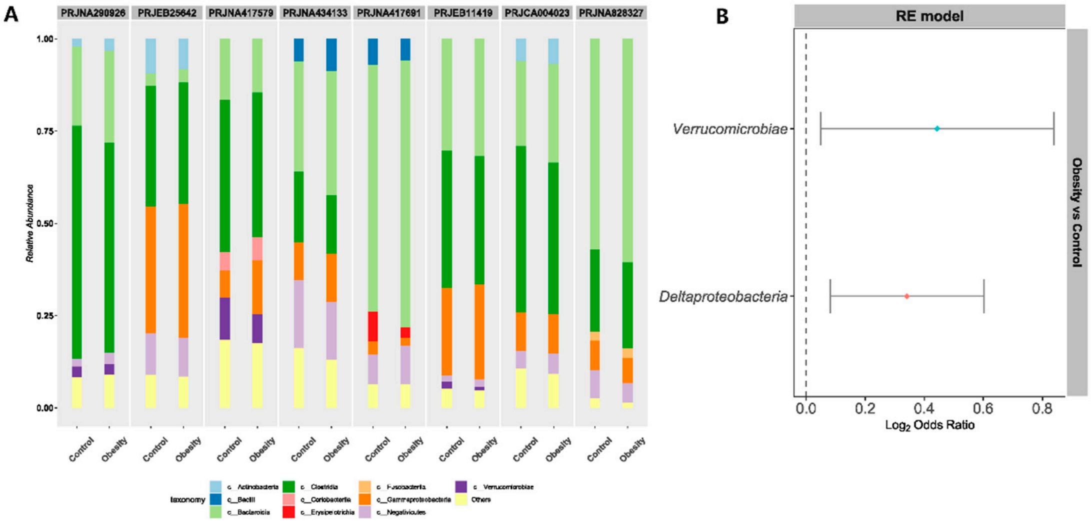

Figure S4. The top five taxa and forest plot at the class level between the obesity and control groups. (A) The top five taxa in terms of relative abundance at the class levels in each study. (B) Forest plot of classes with significant ORs obtained by the random effects model analysis between the obesity and control groups.

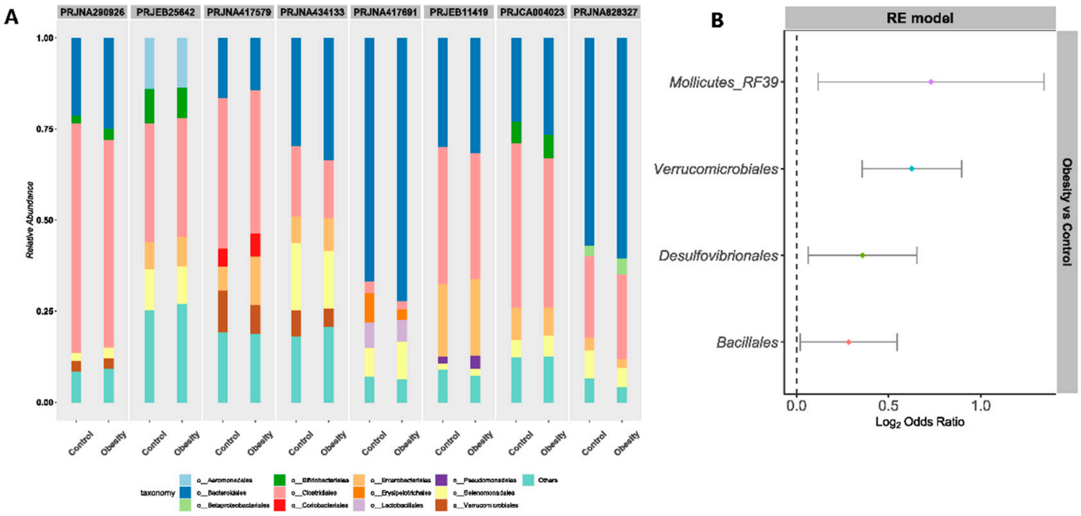

Figure S5. The top five taxa and forest plot at the order level between the obesity and control groups. (A) The top five taxa in terms of relative abundance at the class levels in each study. (B) Forest plot of order with significant ORs obtained by the random effects model analysis between the obesity and control groups.

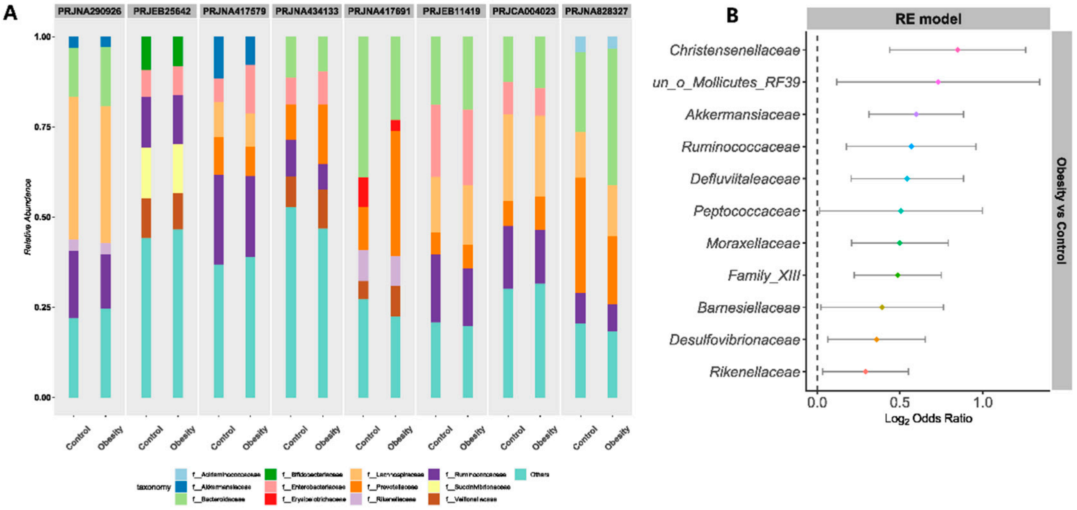

Figure S6. The top five taxa and forest plot at the family level between the obesity and control groups. (A) The top five taxa in terms of relative abundance at the family levels in each study. (B) Forest plot of family with significant ORs obtained by the random effects model analysis between the obesity and control groups.

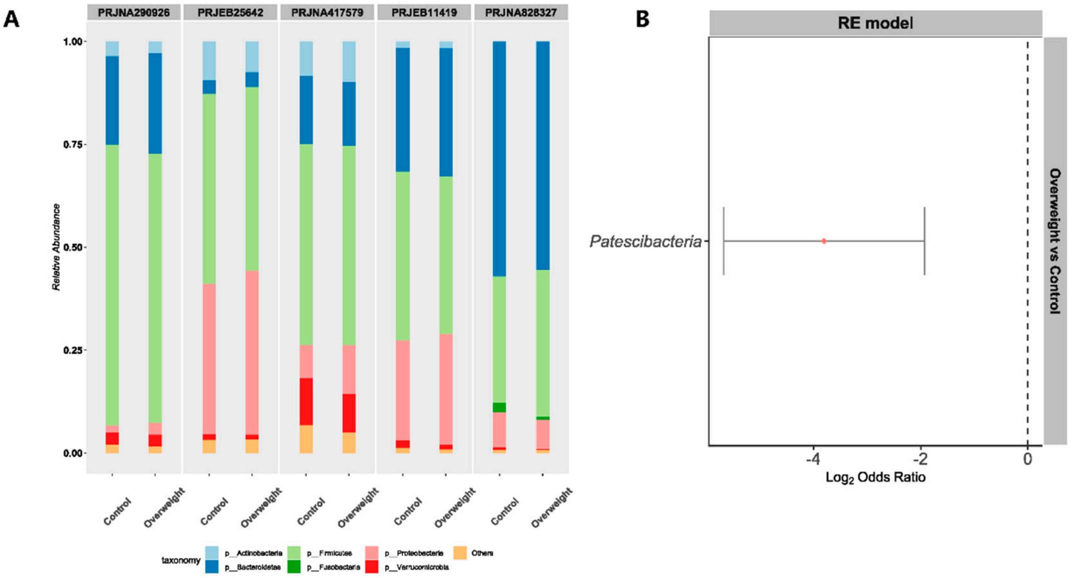

Figure S7. The top five taxa and forest plot at the phylum level between the overweight and control groups. (A) The top five taxa in terms of relative abundance at the phylum levels in each study. (B) Forest plot of phyla with significant ORs obtained by the random effects model analysis between the overweight and control groups.

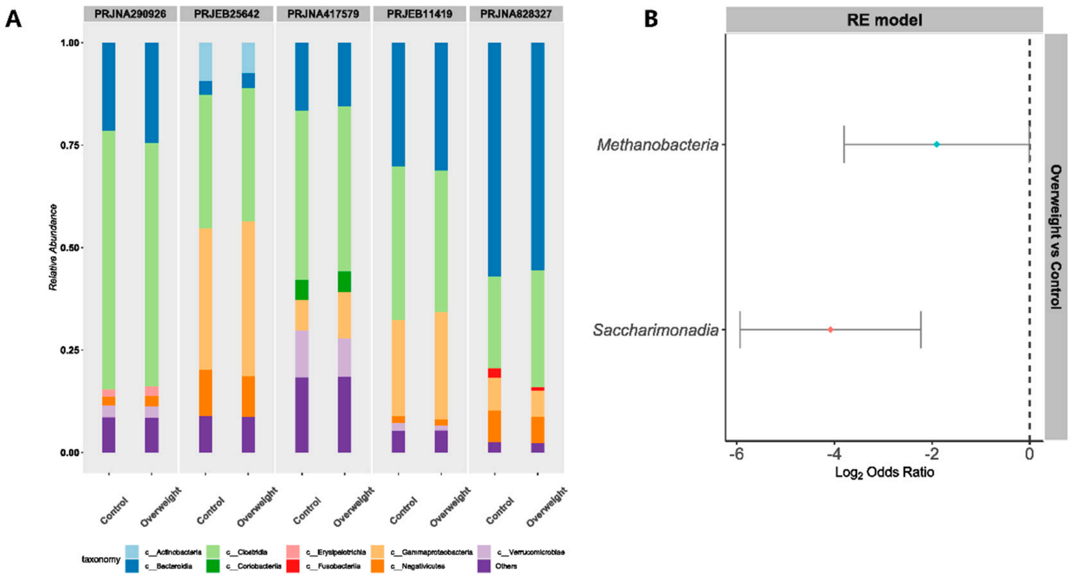

Figure S8. The top five taxa and forest plot at the class level between the overweight and control groups. (A) The top five taxa in terms of relative abundance at the class levels in each study. (B) Forest plot of classes with significant ORs obtained by the random effects model analysis between the overweight and control groups.

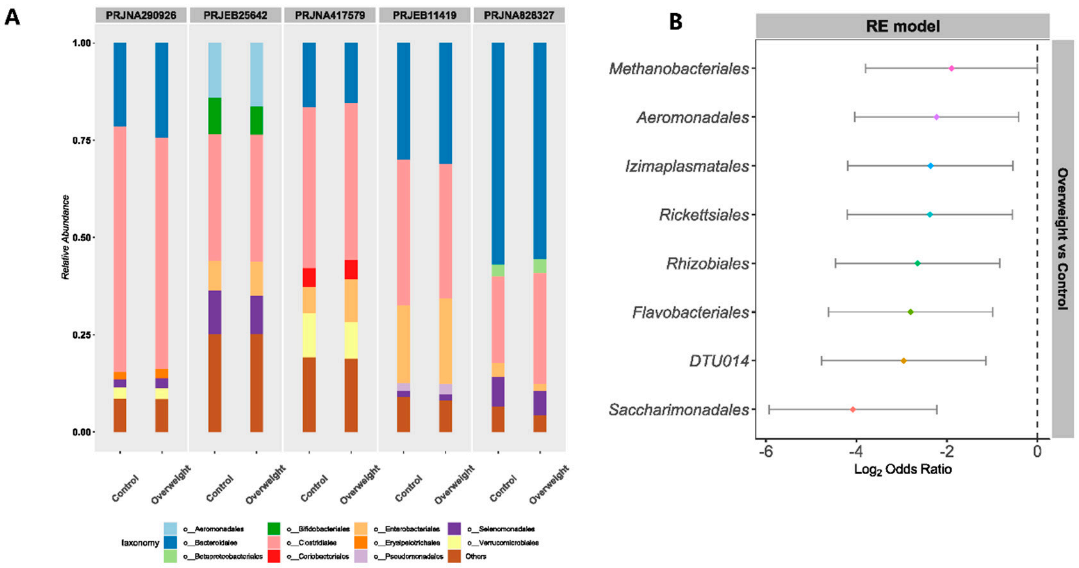

Figure S9. The top five taxa and forest plot at the order level between the overweight and control groups. (A) The top five taxa in terms of relative abundance at the order levels in each study. (B) Forest plot of order with significant ORs obtained by the random effects model analysis between the overweight and control groups.

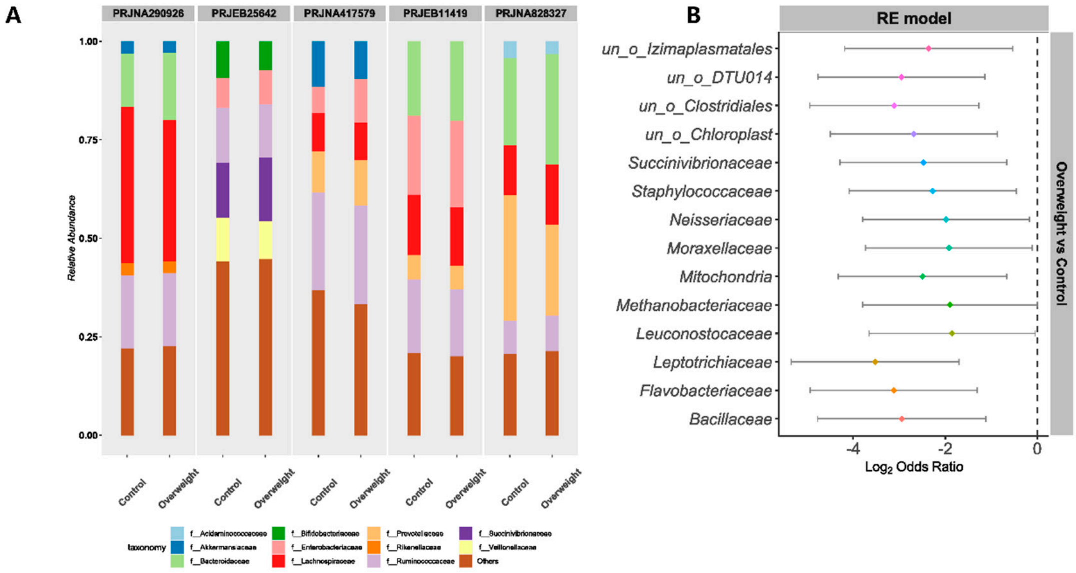

Figure S10. The top five taxa and forest plot at the family level between the overweight and control groups. (A) The top five taxa in terms of relative abundance at the family levels in each study. (B) Forest plot of family with significant ORs obtained by the random effects model analysis between the overweight and control groups.

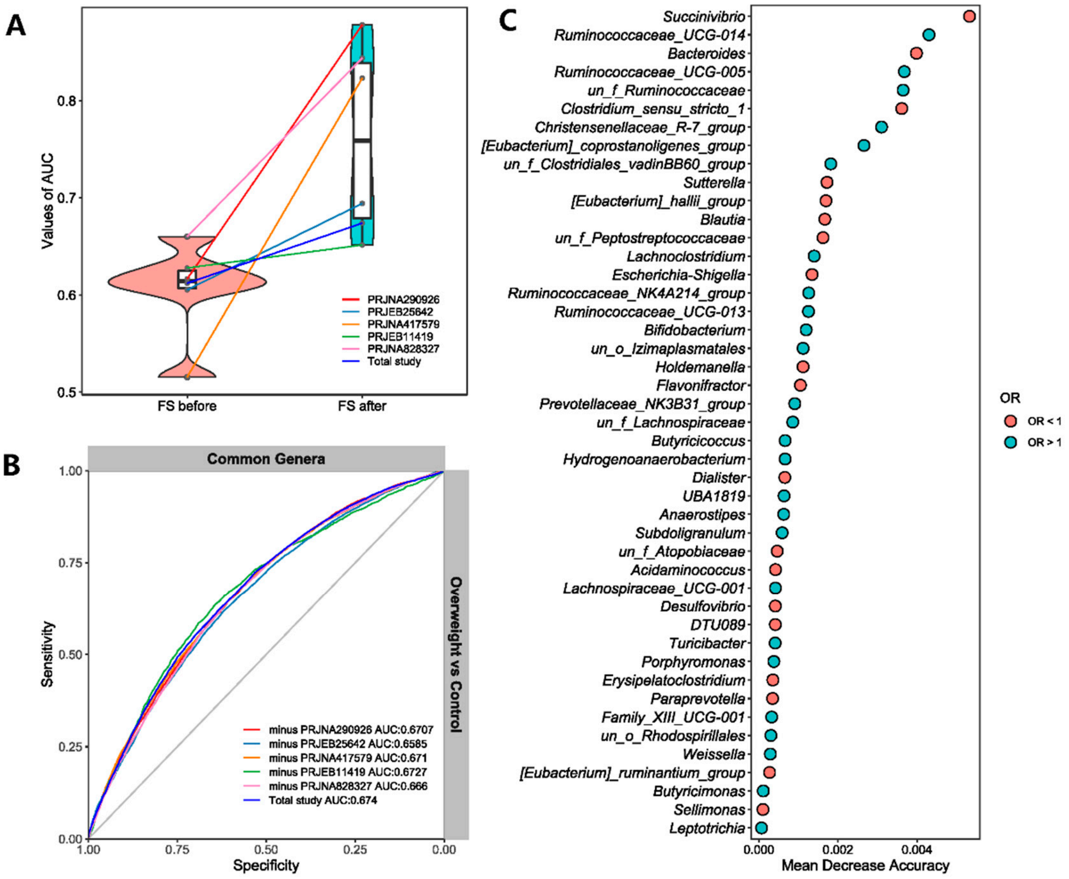

Figure S11. The performances of models to classify the overweight and control groups based on common genera. (A) Improvements in model performances were observed after feature selection (FS). (B) ROC of training data of the leave-one-out study models and training data pooled studies based on selected features. (C) Importance and ORs of features selected in the model based on pooled studies.
