# Supplementary figures and images for: Gut Microbiota Characteristics of People with Obesity by Meta-Analysis of Existing Datasets
Source: Nutrients. 2022 Jul 21;14(14):2993. doi: 10.3390/nu14142993 (PMC9325184; doi:10.3390/nu14142993)

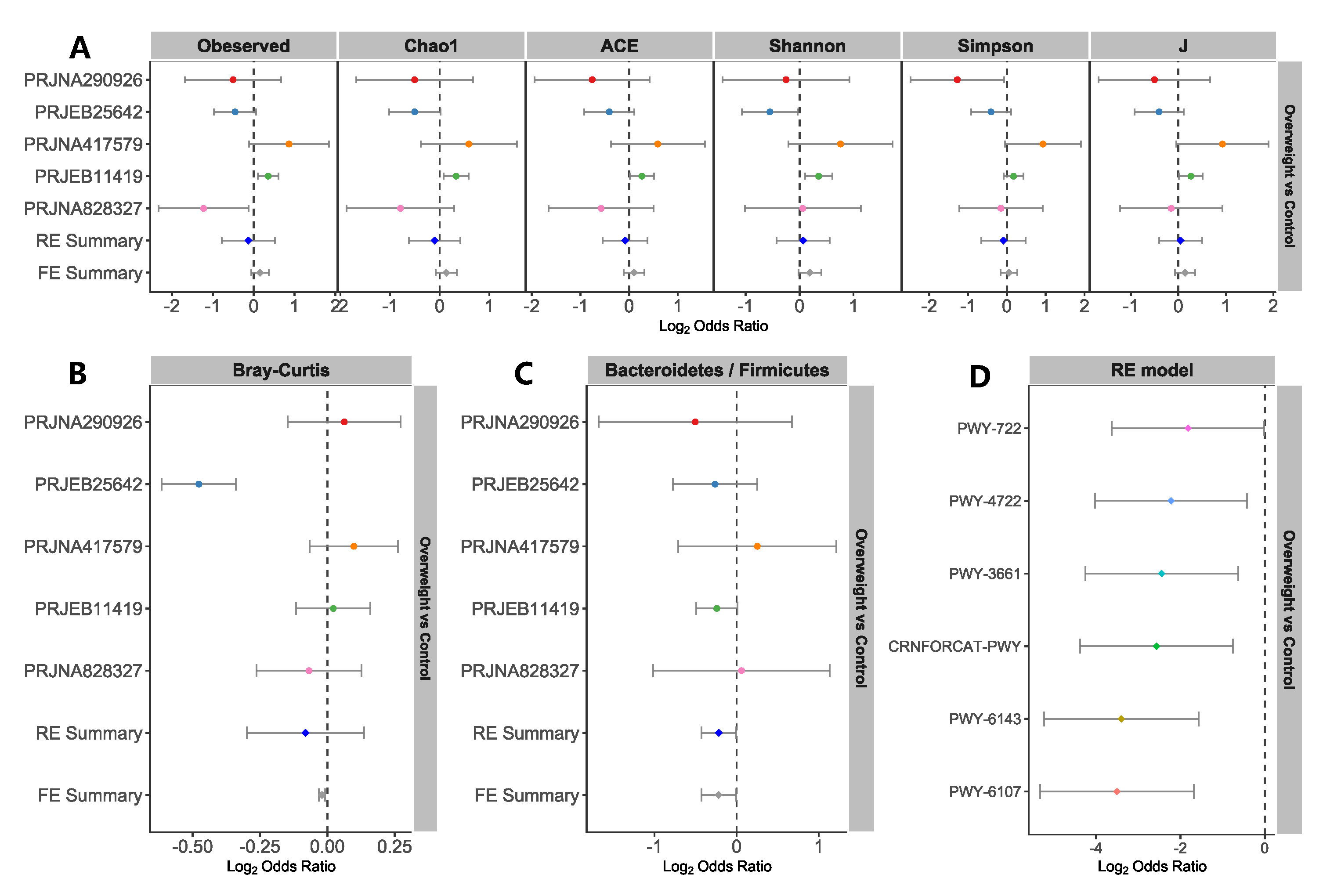

Supplement: Supplementary file 1 [file nutrients-14-02993-s001.zip › Figure S1-11/Figure S1.tif]

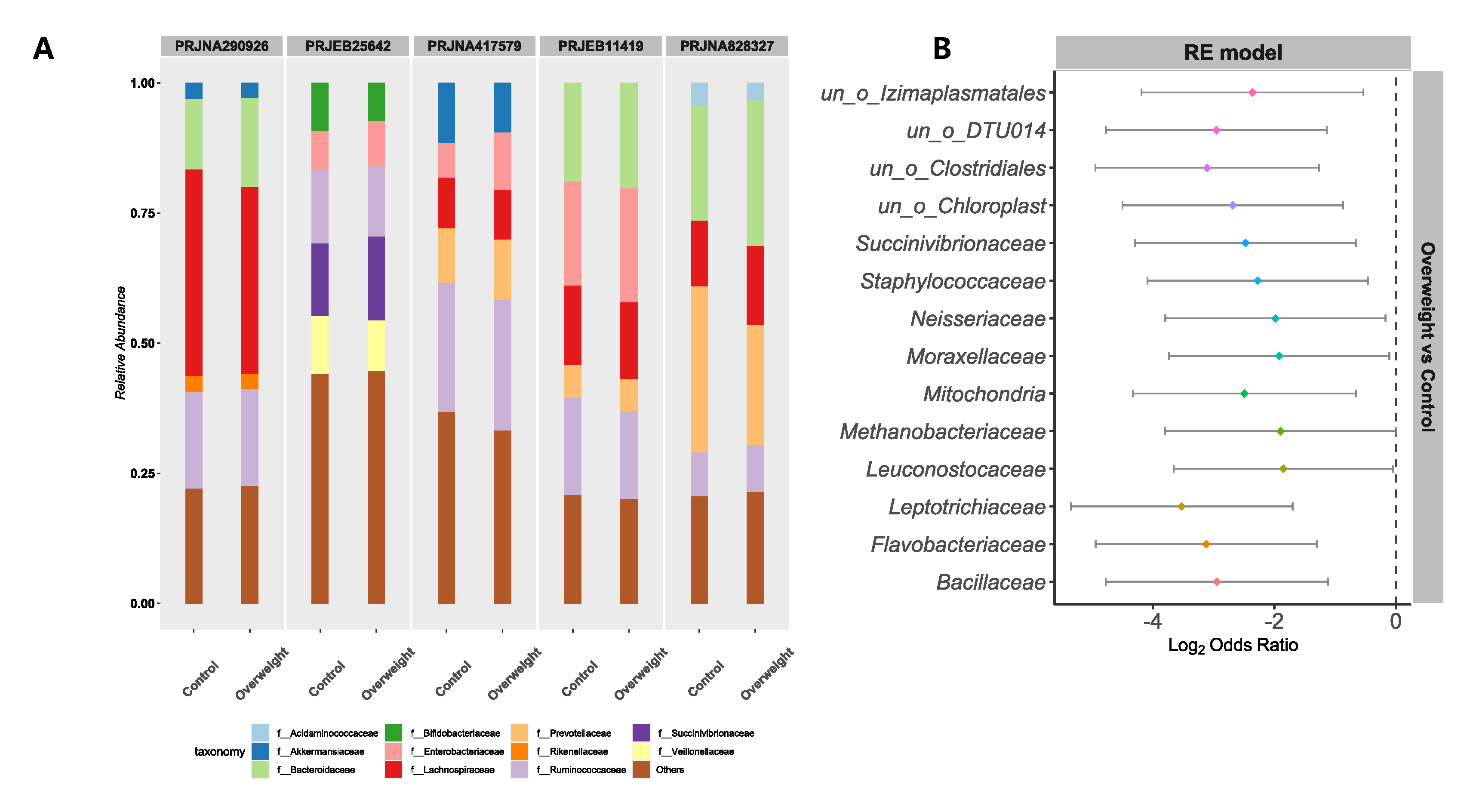

Supplement: Supplementary file 1 [file nutrients-14-02993-s001.zip › Figure S1-11/Figure S10.tif]

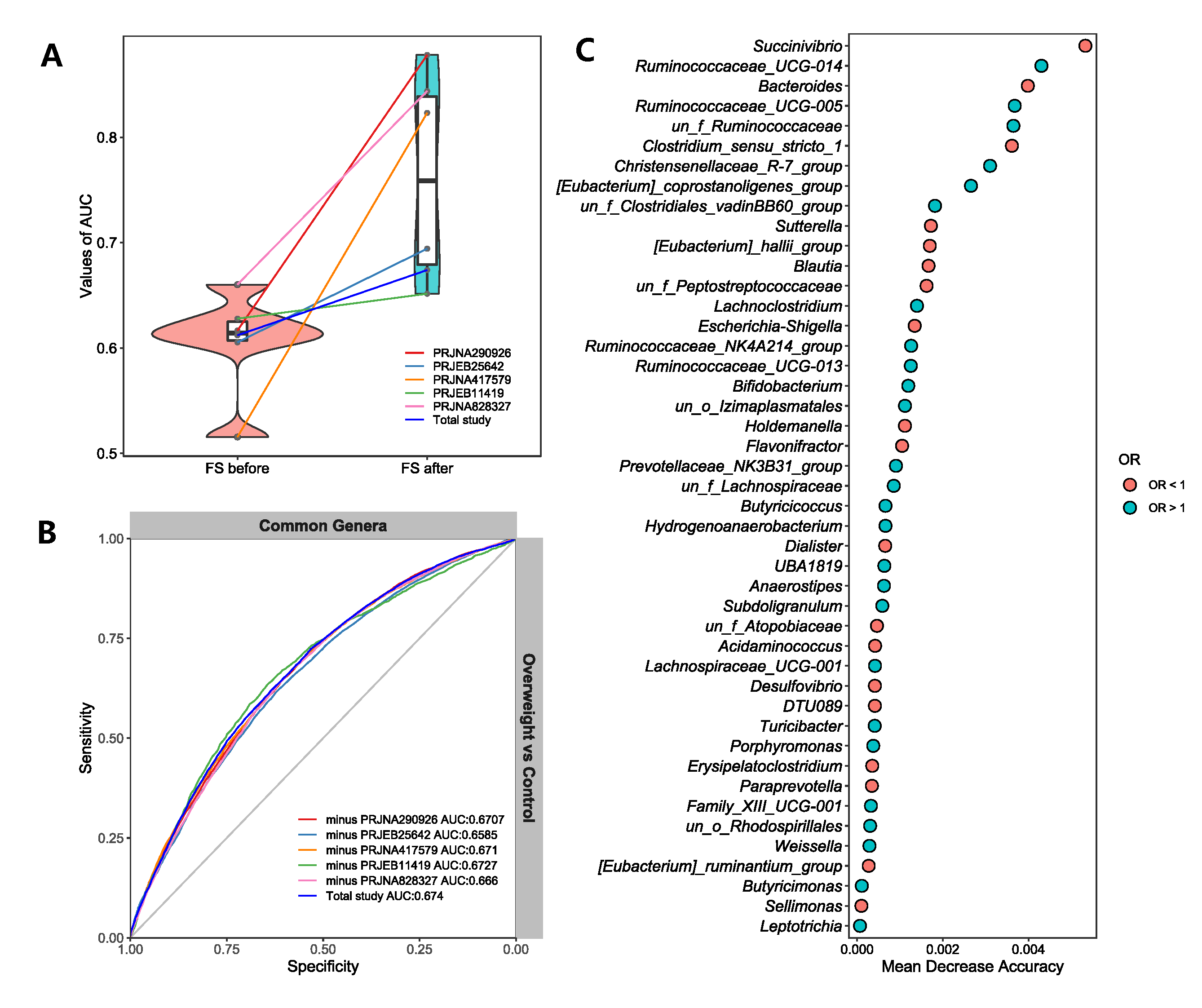

Supplement: Supplementary file 1 [file nutrients-14-02993-s001.zip › Figure S1-11/Figure S11.tif]

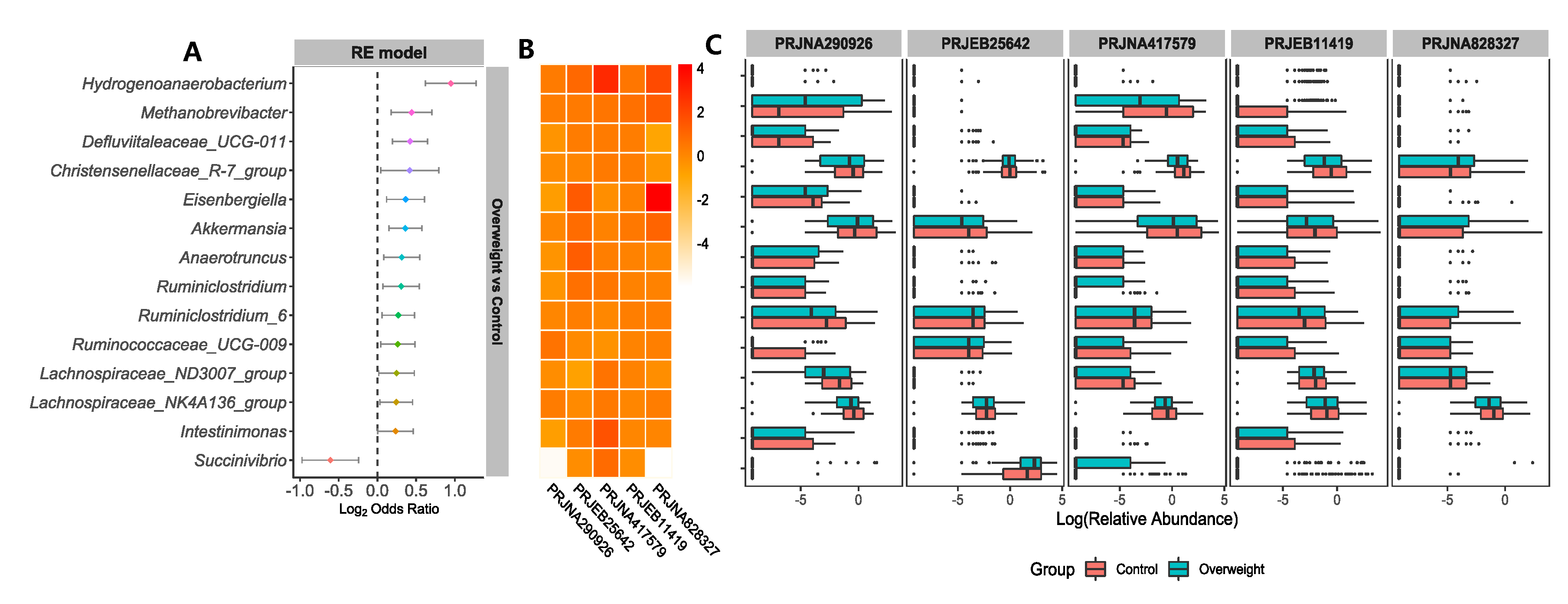

Supplement: Supplementary file 1 [file nutrients-14-02993-s001.zip › Figure S1-11/Figure S2.tif]

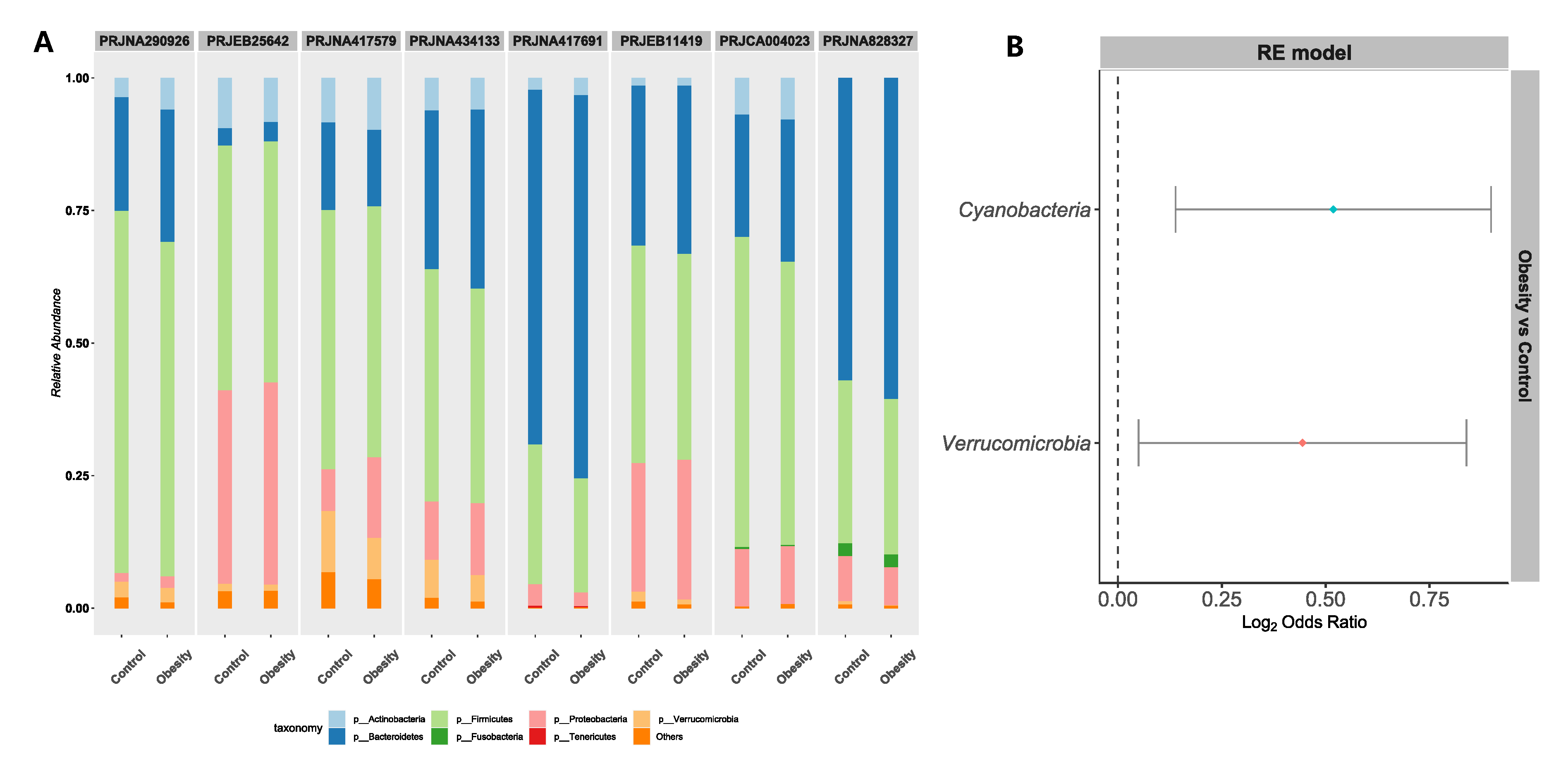

Supplement: Supplementary file 1 [file nutrients-14-02993-s001.zip › Figure S1-11/Figure S3.tif]

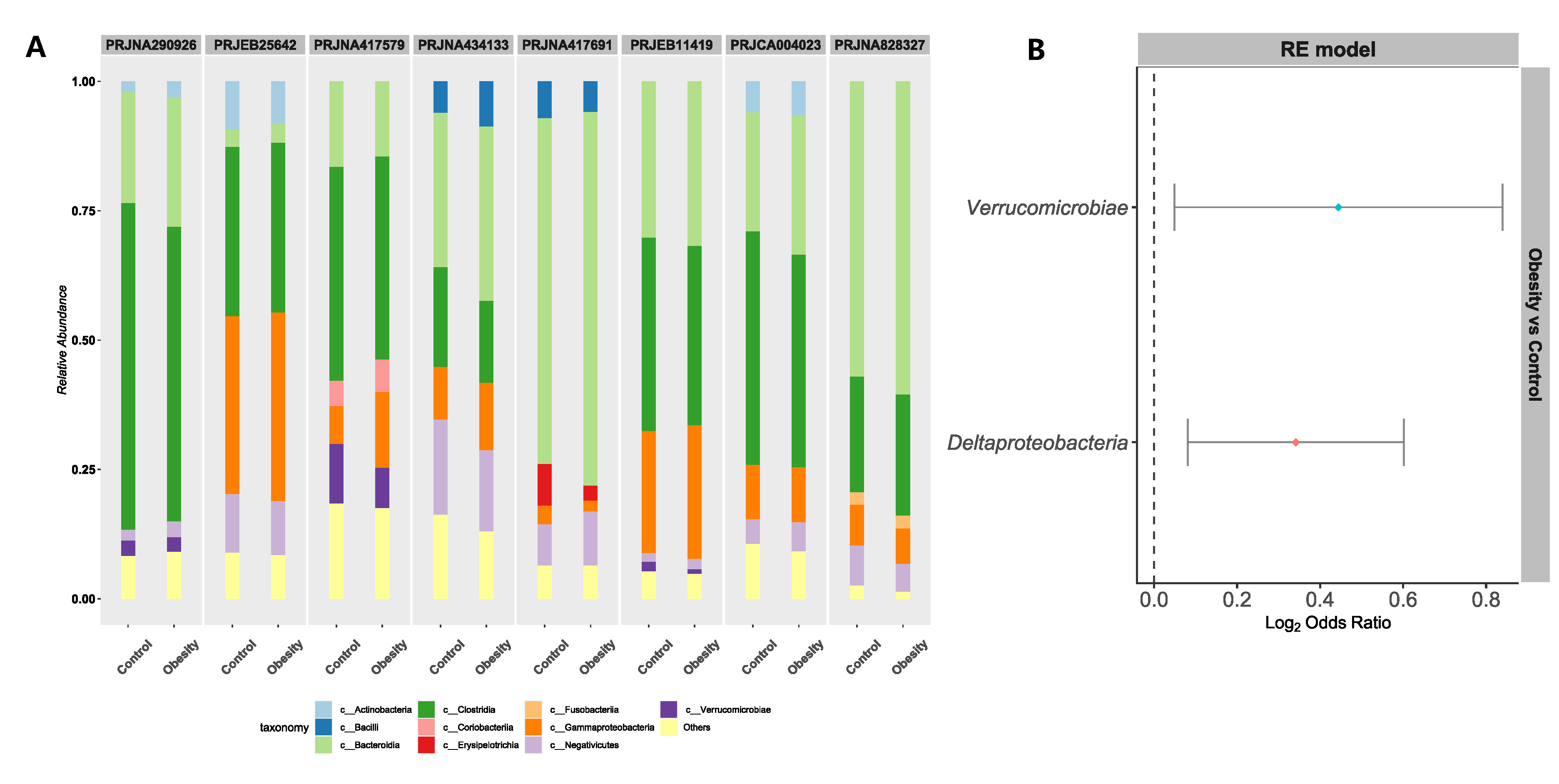

Supplement: Supplementary file 1 [file nutrients-14-02993-s001.zip › Figure S1-11/Figure S4.tif]

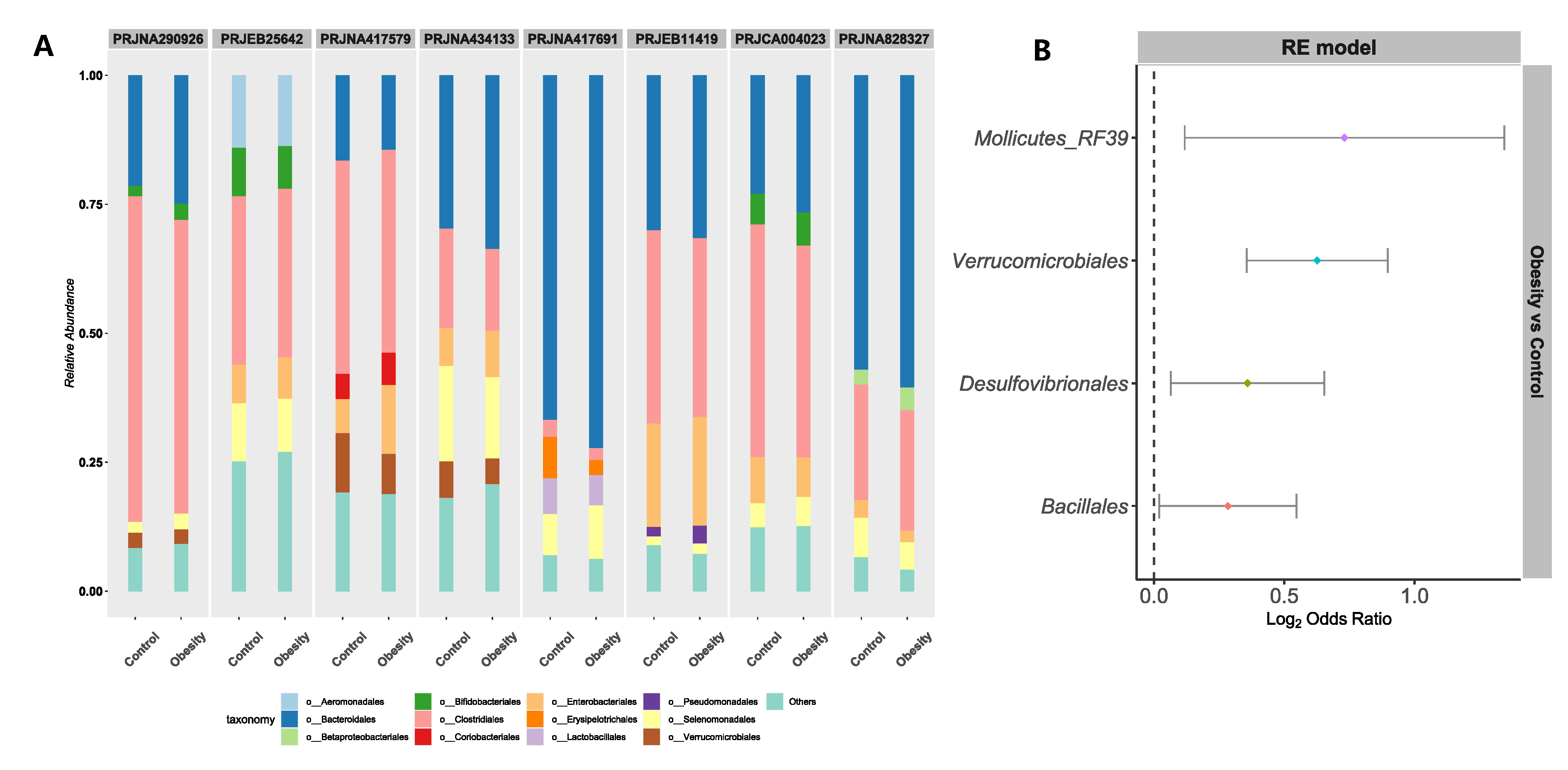

Supplement: Supplementary file 1 [file nutrients-14-02993-s001.zip › Figure S1-11/Figure S5.tif]

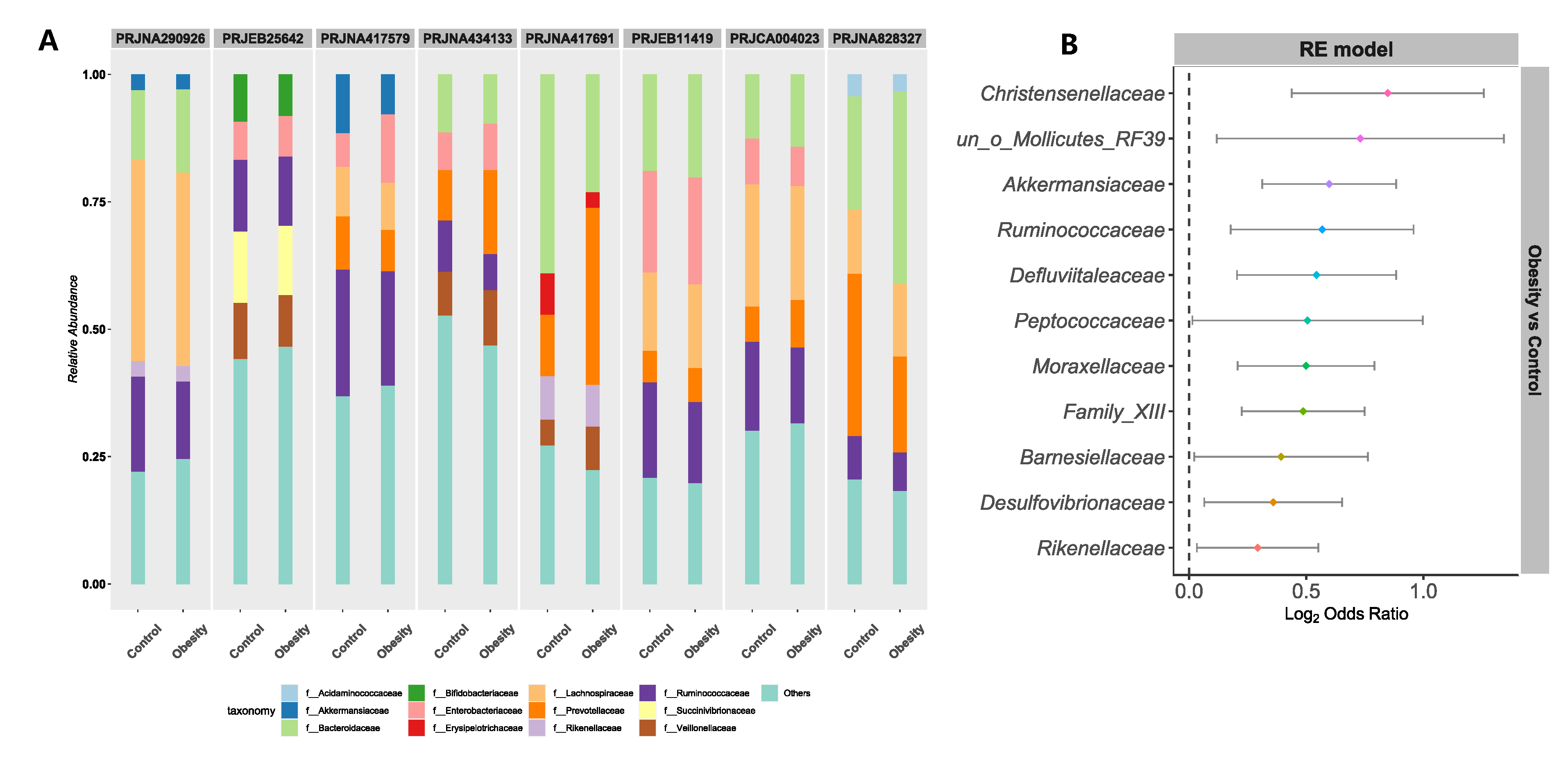

Supplement: Supplementary file 1 [file nutrients-14-02993-s001.zip › Figure S1-11/Figure S6.tif]

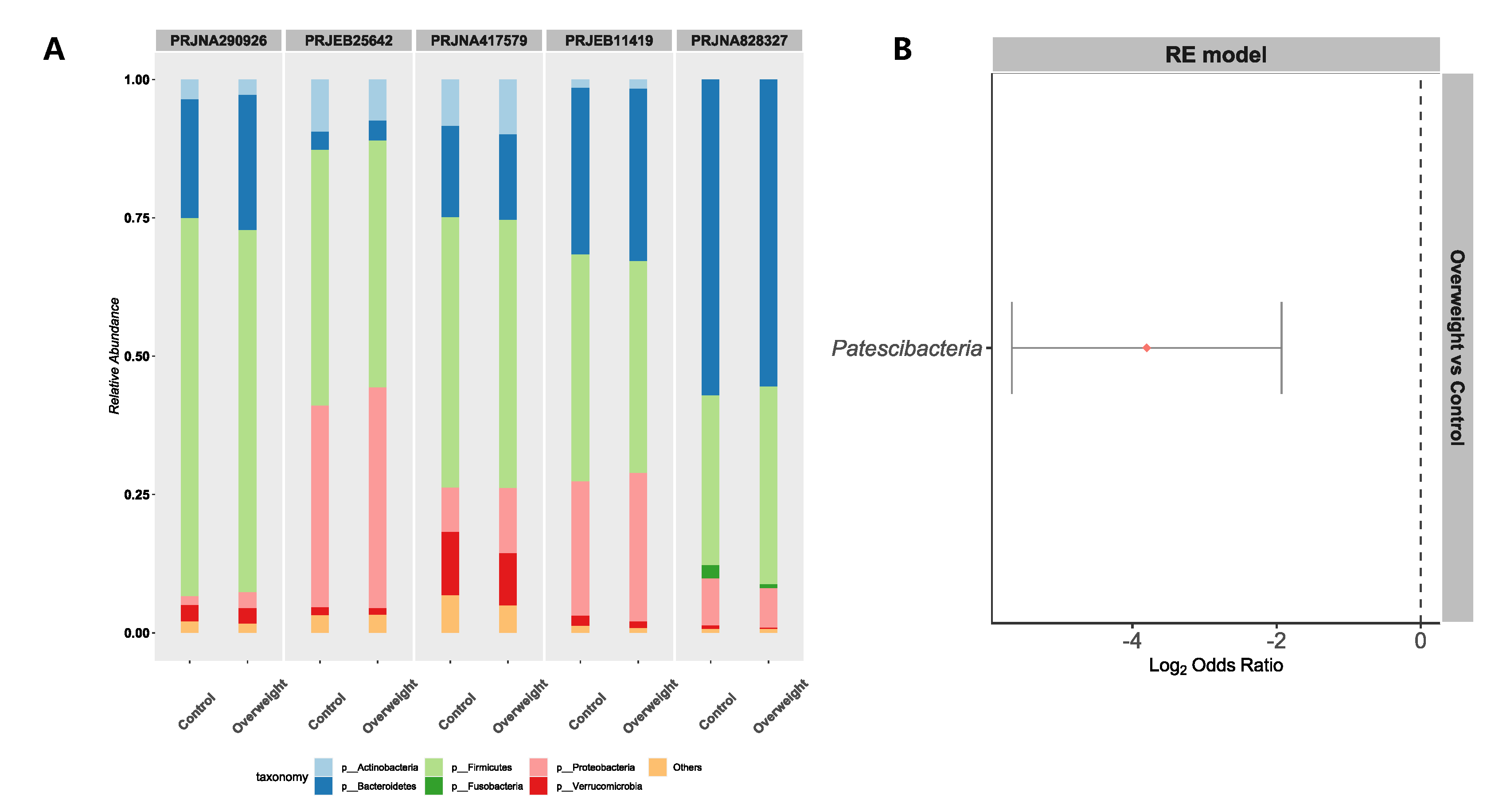

Supplement: Supplementary file 1 [file nutrients-14-02993-s001.zip › Figure S1-11/Figure S7.tif]

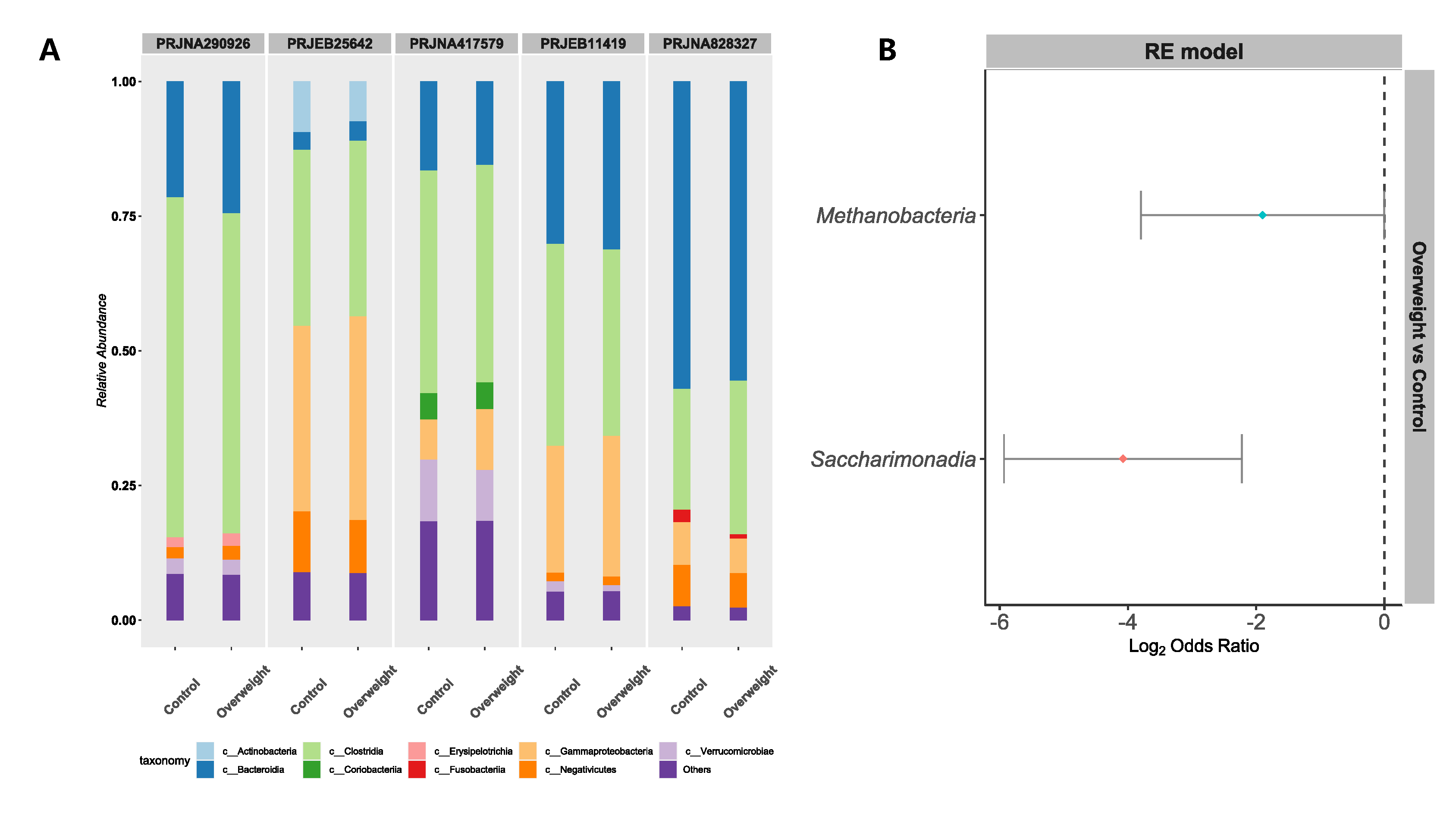

Supplement: Supplementary file 1 [file nutrients-14-02993-s001.zip › Figure S1-11/Figure S8.tif]

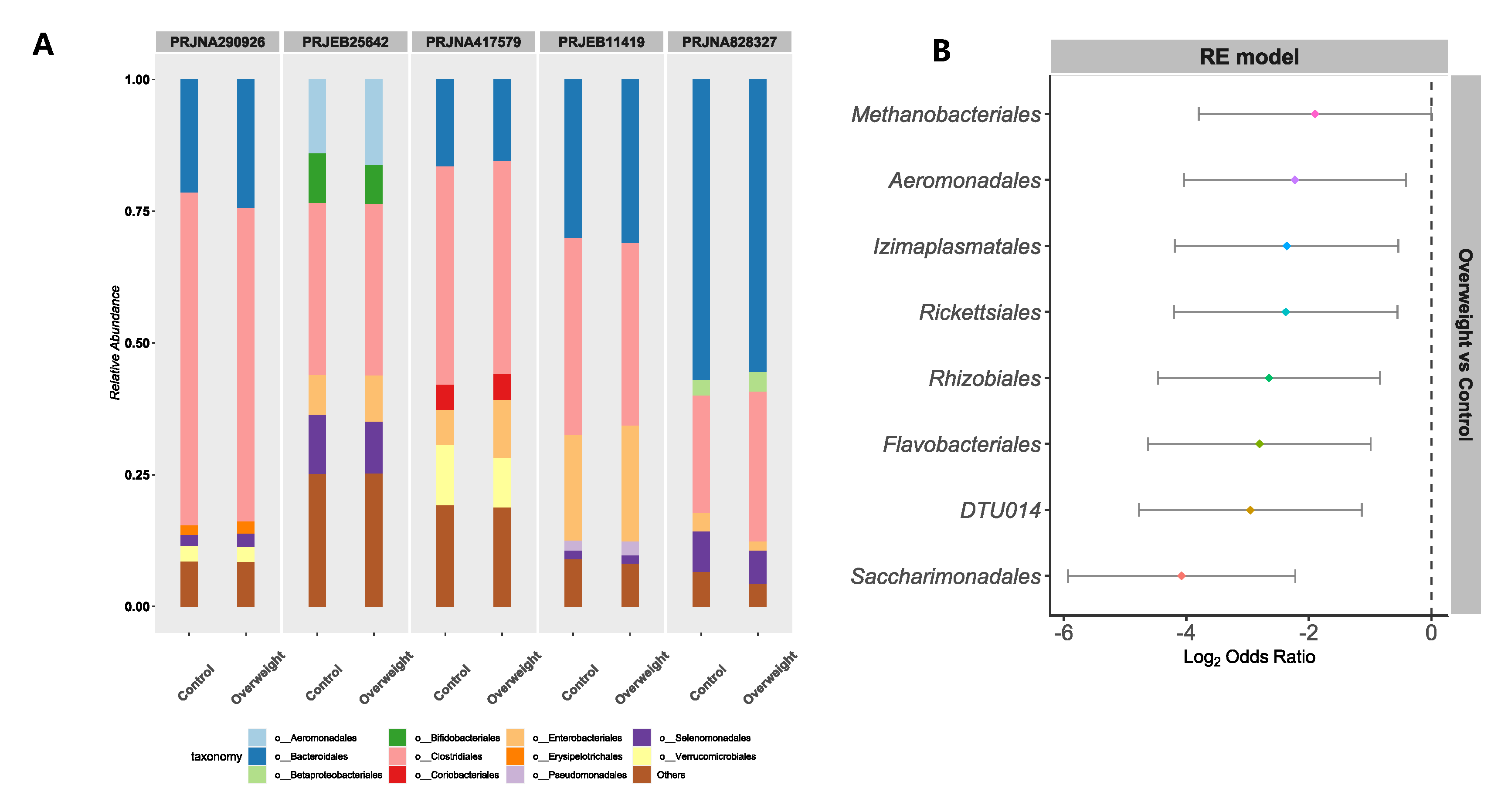

Supplement: Supplementary file 1 [file nutrients-14-02993-s001.zip › Figure S1-11/Figure S9.tif]
